# Famine and food security: new trends and systems or politics as usual? An introduction

**DOI:** 10.1111/disa.12669

**Published:** 2024-11-15

**Authors:** Susanne Jaspars, Luka Biong Deng Kuol

**Affiliations:** ^1^ Food Studies Centre SOAS University of London United Kingdom; ^2^ The Sudd Institute and University of Juba, South Sudan, and Africa Center for Strategic Studies, National Defense University United States

**Keywords:** famine, food security, neoliberalism, politics, war

## Abstract

Over the past decade, famine and food insecurity have increased, yet there have been few articles with a critical analysis of their social and political dynamics. This special issue of *Disasters* aims to revive such analysis and to provide new insights. The special issue contains eight articles, with topics ranging from the role of global politics and neoliberal strategies, to sanctions, war, settler‐colonialism, elite capture and inequalities, actions of resistance and resilience, and the challenges of famine prevention in today's global political context. The papers provide both global and local analysis, with the latter covering Kashmir, South Africa, South Sudan, Sudan, and Syria.

## 1 WHY THE SPECIAL ISSUE?

This special issue of *Disasters* is about the social and political dynamics of famine and food security and different approaches to researching, analysing, and responding to them.^1^ Over the past decade, famine has been firmly on the rise. In 2023, 281.6 million people faced high levels of acute food insecurity in 59 countries/territories, with numbers increasing every year since 2019 (when the figure was 137 million) (FSIN and Global Network Against Food Crises, 2024). Much of this trend is linked to conflict, with severe weather as an added driver. The COVID‐19 pandemic and war in Ukraine have contributed to a rise in global food and fuel prices. Gaza and Sudan are extremes, where famine or the risk of famine have been identified this year (2024). Recognition of increasing food insecurity, famine, and starvation is also evidenced in the number of new global initiatives. This includes United Nations (UN) Security Council Resolution 2417, condemning the starving of civilians as a weapon of war, adopted in 2018, the UN's High‐Level Task Force on Preventing Famine, established in 2021, the UN Food Systems Summit in 2021 and the Global Food Security Summit in 2023, and the G20's Global Alliance Against Hunger and Poverty, proposed in 2024.

Yet, apart from recent work by de Waal and colleagues on ‘mass starvation’ (de Waal, 2018; Conley et al., 2022), the past decade has seen little critical analysis of food security and famine, and few peer‐reviewed articles on these topics. *Disasters* has only published 17 articles on famine since 2000, but 92 in the 20 years before that, and the trend is not limited to just this journal (Rubin, 2019). The reason could be of course a reduction in food crises or famine from the turn of the twenty‐first century, with the number only rising recently. The famine declaration for Sudan (in 2024) is only the fifth in that time frame, with the previous ones being for Somalia (in 2011), Nigeria (in 2016), and South Sudan (in 2017 and 2020). What the articles in this special issue reveal is that over this period, vulnerability to food insecurity has increased as a result of globalisation and neoliberal policies, such as the promotion of free trade and privatisation. Rubin (2019), in addition, argues that the trend of few publications on the subject is due to little interest in theory‐building, issues of access (with most current famines being conflict‐related), or the research community that works on famine being small and dispersed. This special issue is a case in point, encompassing submissions by experts from a wide variety of disciplines: anthropology; development studies; food security; history; politics; and nutrition.

While there have been few articles on famine since the 1990s, *Disasters* has published papers on Eritrea, Ethiopia, Nigeria, Somalia, South Sudan, Sudan, and Yemen in the past decade, all of which experienced acute food insecurity or famine over this period. This raises questions about the conceptualisation of famine and food security, as well as a change in focus towards resilience, safety nets, and protracted crisis, and a need to consider the implications of this shift. From about 2000, there have been articles which reference food or food security, but not famine.

We compiled this special issue to revive discussions about food security and famine and its causes in today's world, and to provide new insights. Has famine changed, or has the way we look at it altered, since the 1990s? The special issue focuses specifically on social and political dynamics. In the past 20 years, the Integrated Food Security Phase Classification (IPC) has become the main tool to determine the severity of food security and famine, relying largely on quantitative indicators of food security, malnutrition, and mortality (Maxwell and Hailey, 2021). However, it cannot also yield in‐depth knowledge of the social dynamics of famine's causation, people's experiences of hunger and famine, or how changes in the global political economy create food insecurity. Furthermore, its governance and leadership structure are dominated by the Global North (FAO, 2022).

In our call for abstracts, we encouraged specifically authors from the Global South, but were only partially successful in this regard. We have a number of articles that are co‐authored by researchers from the Global South and North, but no single authors based in Global South universities or other institutions. This raises wider issues about the nature of academic publications and how to improve access for Global South authors. Another issue is how to link up famine discussions with Global South priorities concerning food sovereignty (see, for example, Fakhri, 2024), although we may have made a start by including a paper on settler‐colonialism and food sovereignty in Kashmir. The majority of the papers in this special issue were first presented during our panel on ‘Food Insecurity and Famine: New Trends and Systems or Politics as usual?’ at the World Conference on Humanitarian Studies on 7 November 2023.

## 2 OVERVIEW

This special issue of *Disasters* contains eight articles that provide an analysis of famine or food insecurity globally and across a range of contexts (Kashmir, South Africa, South Sudan, Sudan, and Syria). The first article looks at changes in global trends that create famine. It is followed by analyses of the effects of various policies, ideas, and actions (local and international) as well as the experiences and strategies of populations affected by food insecurity and famine. The final article considers prevention. They proceed as follows:

1. **Alex de Waal** (2024) analyses the fall and rise of famine, and argues that from 2016, a contest between two global war economies (the United States‐led Pax Americana and the newly‐emerging BRICS^2^ club) creates an environment in which starvation crimes can be committed with impunity.
2. **Mohammad Kanfash** (2024) considers the impact of sanctions and donor conditionality on Syria and shows how they comprehensively undermine the food security of ordinary people.
3. **Tamer Abd Elkreem** and **Susanne Jaspars** (2024) analyse the evolution of power relations in the Gezira agricultural scheme in Sudan, to explore the role of historical, political, and economic processes in the post‐2023 war and famine.
4. **Mehroosh Tak**, **Sardar Babur Hussein**, **Haris Zargar**, and **Lauren J. Blake** (2024) assess the impact of militarised violence and settler‐colonialism on the food system and their role in creating food insecurity in Kashmir, as well as the everyday actions of resistance and resilience.
5. **Khulekani T. Dlamini** and **Elizabeth Hull** (2024) investigate the link between food insecurity, xenophobia, and political legitimacy in post‐COVID‐19 South Africa, and situate it within the wider context of political tensions and social unrest.
6. **Francois Sennesael** and **Harry Verhoeven** (2024) examine the role of environmental narratives in food crises and how political elites in South Sudan imagine the role of the water–energy–food nexus and have used it in consolidating power.
7. **Naomi Pendle** and **Abraham Diing Akoi** (2024) discuss the role of music in understanding the local politics of famine. They show how songs and music shape ‘regimes of truth’ around famine, and who is shamed and held accountable at the community level.
8. **Paul Howe**, **Merry Fitzpatrick**, and **Daniel Maxwell** (2024) review technical and political interventions for preventing famine and suggest and identify five levels of famine prevention.

With three papers on Sudan or South Sudan, it could be argued that the special issue is biased towards these countries. This, however, reflects both the state of famine and food security research and the disproportionate number of famines there. In response to our call, we had more submissions on South Sudan than on any other country—we selected just two that best met the criteria of the request for abstracts. It has been acknowledged before that Sudan (including South Sudan for much of its history) has been a laboratory for developing new approaches to understanding and responding to famine and food insecurity (African Rights, 1997; Jaspars, 2018). *Disasters* has published more than 60 articles on food and/or famine in Sudan (or that include evidence from Sudan or South Sudan) over the years. Many remain influential. These include de Waal's (1988) work on famine early warning and the use of socioeconomic data, Duffield's (1990a, 1990b) political economy analysis of Sudan's emergency and response and the need to move to a general social security system, Keen's (1991) study on the benefits of famine (analysing famine as a process with winners and losers), Macrae and Zwi's (1992) inquiry on the use of food as an instrument of war and Macrae et al.'s (1997) critique of the relief to development continuum, and Howe and Devereux's (2004) research on the famine intensity and magnitude scales that formed the basis of the IPC.

The articles in this special issue not only build on the knowledge created by these authors, but also present new ideas and analysis. Broadly, four themes arise in the submissions. First, the role of global politics, neoliberal economic policies, and volatility in the global food system, creating structural vulnerabilities to food insecurity and famine. Second, how these trends feed into or create inequalities locally, and generate ways for elites to benefit, resulting in different forms of violence that lead to food insecurity. Third, actions of resistance and resilience, and ways of understanding them. Fourth, the challenges of preventing famine in the contemporary context, as well as, simultaneously, the importance of not being complicit in hiding the political acts that cause hunger and famine. Each of these themes is elaborated on below, and of course in much more detail in each of the articles.

## 3 GLOBAL POLITICS AND NEOLIBERALISM‐INDUCED FOOD CRISES

A number of articles in this special issue point to geopolitics and the global economy as playing a key role in creating famine or food insecurity. De Waal (2024) argues that from the mid‐1980s, the neoliberal world order reduced the incidence of famine because of progress in democratic political orders, reductions in poverty, cheap food prices, and an expanded humanitarian apparatus. At the same time, however, structural causes of food insecurity were introduced which ultimately led to greater vulnerability. Neoliberal measures included structural adjustment around the mid‐1980s, emphasising the role of free trade and markets (which has included encouragement of cash crops for export and a reduction in state subsidies) and privatisation. These trends intensified in the 1990s and the 2000s and have been imposed on much of the Global South, leading to national debts (in part owing to the dollarisation of state finance in that part of the world, as de Waal explains). The 1980s also mark the start of a global corporate food regime, with the globalisation of food systems and dominant involvement of multinational corporations within them (McMichael, 2009). The concentration of food trade within a few multinationals, plus profit‐making motivations, and speculation in financialised food systems have been widely recognised as contributing to food crises in 2008 and 2022 (Clapp and Isakson, 2018; Mousseau, 2024).

The effects of these trends are seen across the articles in this special issue. In South Africa, Dlamini and Hull (2024) write that neoliberalism and dependence on global markets have led to increased vulnerability to food insecurity following the COVID‐19 pandemic and global price rises following the war in Ukraine. Structurally, this has fed into racialised legacies of settler‐colonialism and white corporate interests. In Kashmir, Tak et al. (2024) also link neoliberal strategies and settler‐colonialism, this time by the Indian state. A push towards production of cash crops, removal of a minimum producer support price, privatisation of input provision is, they argue, also a tool of economic subjugation, part of the Indian's state political strategy to control Kashmir's population. In Sudan, Abd Elkreem and Jaspars (2024) highlight how neoliberal policies of privatisation enabled profiteering by businesses close to the Islamist regime. This contributed to a deterioration of the country's largest agricultural scheme, in Al‐Gezira, fed into inequalities, and ultimately resulted in the revolution and the military coup, war, and famine. Sennesael and Verhoeven (2024) complement this with an analysis of the motivations of South Sudan's leaders in promoting export‐oriented commercial agriculture—and the building of dams—despite their previous opposition to such projects. This allowed them to control political power but reproduced food insecurity.

As de Waal (2024) notes, ‘from the viewpoint of the Global South, financial rules set in Western capitals veer into instruments of coercion’. Another tool of economic warfare, used by the West, is sanctions. Kanfash (2024) points out how sanctions add to already widespread food insecurity due to war and oppression. Sanctions are used to weaken target states (or individuals) economically and decrease resources to perpetrate violence or encourage elites to concede to foreign demands. They have numerous effects on food security. For example, while not targeting food imports directly, restrictions on Syrian banks and de‐risking measures by international companies mean they are severely constrained. Combined with restrictions on international money transfers, this has led to inflation. Humanitarian carve‐outs were not effective. Similarly, in Afghanistan, Somalia, and Sudan, sanctions led to food insecurity and, in some cases, famine.

Since 2016, famine has become more frequent. According to de Waal (2024), this is partly because of the changing nature of global war economies. The formation of the BRICS club, in 2009, with a key aim of de‐dollarisation, challenges US hegemony and global neoliberal economics. The members' need to underpin currency with gold, land, or oil has wreaked havoc on countries like Sudan, particularly after Egypt, Ethiopia, Iran, and the United Arab Emirates joined in 2024—Saudi Arabia was also offered membership. Combined with their rejection of multilateralism and humanitarianism as a tool of the West, this trend has consolidated extractive war economies, especially those on the fault lines of Western and BRICS power struggles. This is where political, economic, and security concerns trump humanitarian ones. We see this played out in the case studies in this special issue, and of course in Gaza, through the effect on power relations and various acts of violence.

## 4 THE ROLE OF LOCAL ELITES, INEQUALITIES, AND ACTS OF VIOLENCE

Global political and economic processes have fed into, exacerbated, or created local inequalities and power relations, in some cases leading to war and famine and in others to less visible forms of ‘slow violence’ (Nixon, 2013) against particular population groups. These processes generate power for some, and vulnerability to food insecurity among others, and often involve different aspects of production, trade, and aid. In Syria, for example, sanctions contributed to the informalisation of the economy and the establishment of an opportunistic and politically‐connected business elite (Kanfash, 2024).

In Sudan, the Gezira scheme, discussed by both Abd Elkreem and Jaspars (2024) and Sennesael and Verhoeven (2024), provides an example of a large‐scale, externally‐driven commercial agricultural scheme that ultimately impoverished farmers, pitted tenants against labourers, and enabled those close to former regimes to benefit. Neoliberal policies, along with the politics of patronage, played a key role here. These tensions and inequalities continue to play out today, in 2024, feeding into the war and creating famine (Abd Elkreem and Jaspars, 2024). More generally, external actors and policies have fed into Sudan's military–security–industrial complex that controls the country's key resources, including agriculture, fuel, and gold, as well as banking and trade (Gallopin, 2020), and which the army and para‐military Rapid Support Forces are now fighting over. Environmental narratives have reinforced technocratic approaches to food security and contributed to elite capture. For instance, when southern Sudan was under the government in Khartoum, the capital of Sudan, the Sudan People's Liberation Movement contested the building of dams, viewing them as exploitative and hunger‐producing. However, in government, it promoted a water–energy–food nexus and associated environmental narratives as a natural approach to state‐building and used them to consolidate power (Sennesael and Verhoeven, 2024). Globally, environmental narratives hide the role of commodity speculation on global food markets in causing crisis.

Articles on South Sudan, Sudan, and Syria also illustrate the proximate causes of food insecurity and famine. These include direct destruction, killing, and displacement, as well as siege tactics and the use of starvation as a weapon of war (for example by attacks on agriculture, theft of food and livestock, and blocking trade). As Keen (1991) points out, these acts of famine creation can yield benefits: from selling food at high prices, to buying livestock and labour at low ones. The denial or diversion of food aid has been part of this (Jaspars, 2018). These acts are usually aimed at specific population groups—often those already historically marginalised. In Somalia, for example, the Bantu and Rahanweyn, located in south‐central regions, have repeatedly suffered famine (Maxwell and Majid, 2016), as have ethnic groups like the Masalit in western Sudan before and at the time of writing (Human Rights Watch, 2023). Others associated with the rebellion are similarly targeted, and from mid‐2024, populations in El‐Fasher and nearby camps, are under siege. As Abd Elkreem and Jaspars (2024) illustrate, the weaponisation of communications networks has now become part of the political economy of war, famine, and aid.

In addition to these forms of extreme violence and deprivation of objects essential to survival, we see what can be called forms of ‘slow violence’ (Nixon, 2013). That is, violence that occurs gradually and that involves seemingly small and less visible acts of deprivation and starvation, but which build up over time. Tak et al. (2024) employ this concept in relation to Kashmir, where the military occupation undermines people's livelihoods in a number of ways, which, combined, lead to reduced access to food and to malnutrition. These acts include the reduction of land available for cultivation (through the establishment of military infrastructure) and restriction of movement (owing to the military presence), hindering agricultural production, fishing, and the collection of forest products, and necessitating the relocation of kitchen gardens due to women's fear for their own safety. These in turn result in increased dependence on Indian subsidised food distribution and food imports, both of which change local food systems and are a means of controlling the population. Similarly, it could be argued that the treatment of labourers on Sudan's commercial agricultural schemes were acts of slow violence: through the lesser provision of services, shelter, and citizenship, as compared to tenants. Such frequent and continuous acts of violence can be seen to a much greater extent in the Occupied Palestinian Territories, including the West Bank (O'Callaghan, Jaspars, and Pavanello, 2009), where restrictions on freedom of movement and access to land result in food insecurity; and, in 2024, turning into large‐scale direct acts of violence too.

In South Africa, this kind of slow violence took the form of failure to consider the food security impacts of lockdowns during the COVID‐19 pandemic, uneven support eventually, and apparent profit‐making by supermarkets. It contributed to political unrest (including the looting of shops) that challenged the government's political legitimacy. In addition, it led to the blaming of migrants for the crisis (from elsewhere in southern Africa) or to people blaming themselves for not being self‐reliant enough (Dlamini and Hull, 2024).

## 5 RESISTANCE, RESILIENCE, AND SHAME

The articles in this special issue reveal the different ways in which people make sense of and navigate situations of food security, and how they experience it or take action individually or collectively. Submissions on Kashmir, South Africa, South Sudan, and Sudan, for example, refer to different forms of resilience and resistance by people faced with acts that undermine their access to food. They sometimes include discussion of how becoming self‐reliant is perceived as something that is an individual responsibility (South Africa), and how there is a sense of shame if they are not able to ‘cope’ with famine or if they are unable to support others and prevent famine deaths (South Sudan).

In Sudan, Abd Elkreem and Jaspars (2024) describe how tenant farmers and labourers in the Gezira scheme became involved in different forms of resistance, with tenants forming Farmers' Associations (as unions had been co‐opted by government), and labourers organised to form the *Canabi* (camps dwellers) Congress. Both were part of the revolution that deposed the Islamist government of President Omar Al‐Bashir in 2019, but when the new transitional government could not meet their demands, they joined opposing forces. The tensions between them continue to be manipulated during the current war. In South Africa, the looting of supermarkets and other forms of social unrest, which ultimately challenged the legitimacy of the national government, are also forms of political resistance. In Kashmir, under strict military occupation, such political resistance is not possible. In this case, Tak et al. (2024) argue that resilience becomes a form of resistance: actions to continue to produce and share local food, and thus retain a degree of independence or food sovereignty, defying the strategies of the Indian government that promote dependence on imports and state subsidies. Similar acts of resistance can be seen in Gaza, where preparing indigenous food while being starved is about retaining a relationship with land, territory, and history, as well as a means of expressing one's dignity and self‐determination (Fakhri, 2024).

Pendle and Akoi (2024) set out different forms of resistance again, this time through music. Via song, sensitive issues concerning the creation of famine can be brought out into the open, such as the role of soldiers. Song has also been used, however, to shame people who did not help kin or neighbours, when it was thought they could have done so, or people who fled. This shame has the potential to last for generations. In South Africa, Dlamini and Hull (2024) suggest that food‐insecure people, who felt that they had not been able to look after their family sufficiently, sometimes blamed their lack of education and poor employment for this, rather than the structural causes highlighted above. These examples also illustrate how resilience ideology and the shifting of responsibility from international or national actors to individuals can become internalised; responsibilisation being a tactic of neoliberalism. While resilience approaches are still widely promoted as a way of addressing a protracted crisis or vulnerability to one (see Howe, Fitzpatrick, and Maxwell, 2024), these approaches have also been widely criticised for failing to take account of power relations or structural causes of food insecurity and transferring blame to the victims (see, for example, Howell, 2015; Joseph, 2016; Duffield, 2018; Jaspars, 2021). At worst, shifting blame has the potential to dehumanise people who are starving, blaming the hunger on the victims or their political leaders, or by appealing to a higher set of values—and thus aiding the perpetrators of mass atrocity (de Waal, 2024).

## 6 CHALLENGES FOR FAMINE PREVENTION

With this special issue of *Disasters*, we aim to move beyond the technocratic approaches that have come to dominate food security and famine analysis. Rather than using quantitative indicators like dietary diversity and food consumption scores, or classifying the severity of food insecurity using quantitative measures of malnutrition and mortality, the compilation provides a range of political, anthropological, and other qualitative approaches that helps to analyse the social and political dynamics of food crisis and famine.

Any analysis of famine and food insecurity needs to consider social, economic, and political dynamics; both structural and proximate causes. All of the articles in this special issue show the long historical trajectory of famine and hunger creation and its importance for understanding who is vulnerable to famine and why, and how food insecurity and famine are created and by whom. Sennesael and Verhoeven (2024) underline the importance of understanding the discourses around the causes of a food crisis, its origins, and its political effects. The discourse on resilience is another case in point (see also Jaspars, 2021). Other articles (on South Africa, South Sudan, and Sudan) emphasise the value of in‐depth ethnographic fieldwork to understand local power relations and people's experience of hunger. Dlamini and Hull (2024) and Pendle and Akoi (2024) illustrate the relevance of focusing on the human dimension, experience of food insecurity and hunger, and how the latter reflects the various dimensions and causes of food insecurity. Tak et al. (2024) and Abd Elkreem and Jaspars (2024) demonstrate how these methods can be adapted in highly politically‐sensitive situations by relying more on observation, including transect walks. Tak et al. (2024) also present a food systems approach as a way of examining the political economy of food. A spotlight on national politics and international policy needs to be complemented by attention to the detailed political realities on the ground and the contexts in which famine occurs.

Howe, Fitzpatrick, and Maxwell (2024) present a framework for famine prevention, consisting of five levels. This is useful because it outlines, in theory, what is needed to prevent famine. Given the political dynamics discussed in this special issue, it is also not surprising that there are enormous challenges to doing so. As expected, in reality, famine prevention largely centres on altering or preventing proximate risks and mostly involves technical interventions. Anticipating and averting famine would require changes in the global political (or war) economy and in a corporate and financialised food system that profits from food crises and contributes to climate change. These are not issues that even the UN's High‐Level Task Force on Preventing Famine, Special Envoys to Gaza and Sudan, or the Special Rapporteur on the Right to Food can address easily. Famines in Gaza and Sudan show the difficulties faced by humanitarian organisations in preventing proximate famine risks or even responding to them. Indictments by the International Court of Justice and seeking an arrest warrant by the International Criminal Court in the case of Gaza, in addition to reports by the Food and Agriculture Organization and the World Food Programme and to the UN Security Council (FAO and WFP, 2024), are having little or no effect on the conduct of war and its humanitarian consequences. Instead, what we see is different standards being applied, depending on political priorities, to hold warring parties to account under international law. While the hope has to be that famine can be prevented, as proposed by Howe, Fitzpatrick, and Maxwell (2024), a more realistic future is that predicted by de Waal (2024), based on Gaza and Sudan, where ‘the most vulnerable are set to become victims in yet another turn of the global war economy’.

To come back to the question in the title of this article, some global trends in famine and food security causation are new, but they are also continuations of historical processes that are very much political in nature. Perhaps technocratic approaches to identifying and responding to famine, developed in the past two decades, have led to fewer publications presenting a critical analysis of its social and political dynamics. This must be reversed. Even given the enormous odds of preventing famine, what humanitarians and researchers can do is to make sure that the structural causes of food insecurity and famine are understood—not hidden among numbers or discourse, and not forgotten.

## FUNDING STATEMENT

We would like to thank the Global Network Against Food Crises for part‐funding the production of this special issue.

## Data Availability

Data sharing is not applicable to this article.

## Endnotes

^1^
The production of this special issue was part‐funded by the Global Network Against Food Crises.

^2^
BRICS stands for Brazil, Russia, India, China, and South Africa. On 1 January 2024, Egypt, Ethiopia, Iran, and the United Arab Emirates also joined the club and Saudi Arabia was offered membership.
